# Body Mass Index and Musculoskeletal Pain: A Cross-Sectional Study

**DOI:** 10.7759/cureus.13400

**Published:** 2021-02-17

**Authors:** Susana Rosa, Diogo Martins, Mariana Martins, Bruno Guimarães, Leonor Cabral, Luís Horta

**Affiliations:** 1 Physical Medicine and Rehabilitation, Centro Hospitalar Universitário de Lisboa Central, Lisbon, PRT; 2 Physical Medicine and Rehabilitation, Central Entre o Douro e Vouga Hospital, Santa Maria da Feira, PRT

**Keywords:** obesity, musculoskeletal, pain, morbid obesity

## Abstract

Introduction

With obesity reaching pandemic proportions, its detrimental influence on many health-related conditions has recently become a focus of research. Musculoskeletal (MSK) pain is one of several disorders associated with obesity.

Materials and methods

This study was designed to identify MSK pain in individuals with severe obesity, recruited from a specialized obesity clinic, and to evaluate the correlation between the MSK symptoms and some individual criteria.

Results

In 466 patients (342 females and 124 males), with a mean BMI of 42,77 ± 5.64 kg/m², 90,3% reported MSK pain. Severe pain was reported by 57,8% of female *vs* 26,6% of male patients. Female patients showed higher mean pain level when compared with males. Body sites with a higher prevalence of pain were: knee (63.5%), lumbar region (46.8%), hip (29%) and ankle (23%), with a predominance of severe pain. BMI have shown a positive correlation to numeric rating scale score in female participants in three body regions: shoulder (P = 0.010), knee (P = 0.042) and ankle (P = 0.024). Body sites with higher pain prevalence were especially in areas of increased mechanical load.

Conclusions

Understanding the relationship between obesity and pain may provide insights into preventive measures and therapeutic strategies for MSK pain. Persistent MSK pain is a significant problem in obese patients that can have a negative relationship with quality of life and function. This fact highlights the importance that patients with obesity may have access to interdisciplinary care, for the prevention and rehabilitation of MSK disorders. To further understand this association, more robust studies are needed.

## Introduction

Obesity is yet a neglected public health concern. It is a chronic disease, with multifactorial genesis, requiring concerted efforts, posing as an important risk factor for the development and deterioration of another diseases [1]. Worldwide obesity has nearly tripled since 1975. In 2016, more than 1.9 billion adults were overweight. Of these, over 650 million were obese [2].

In 2015, the National Institute of Health Doutor Ricardo Jorge conducted a study that described the prevalence of overweight and obesity in the Portuguese population, verifying that 38.9% of the adults (25-74 years) living in Portugal was overweight and 28.7% suffered from obesity. The prevalence of overweight was higher in men (45.4%), while the prevalence of obesity was higher in women (32.1%) [3].

MSK pain is one of several disorders associated with obesity [4]. Mechanisms linking obesity and pain are complex and include behavioral, mechanical, biological and genetic factors. Pain affecting weight-bearing joints such as the knee, ankle and foot, as well as back pain, are among the most frequent complaints, often reported together as multisite pain [5,6]. Excessive weight increases mechanical stress to the joints and tissues of the body [7] and induces physical limitations and bodily pain. Self-reported bodily pain symptoms increase with progressively higher body mass index (BMI) values [8].

Reported risk factors for MSK pain include older age, female gender, high occupational workload and low physical activity [9-12]. Obesity is also closely related to MSK pain and physical dysfunction [13,14]. Specifically, increasing severity of pain is observed at higher BMI classifications [15,16].

The authors collected and detailed all the data of a sample of individuals with morbid obesity followed in an obesity and metabolic diseases unit. Additionally, the MSK pain was investigated in this group of patients, to evaluate the relationship between BMI and MSK pain.

## Materials and methods

The authors conducted a cross-sectional study (data collected between 2017 and 2018). The patients were recruited from a specialized Obesity Clinic, named “Surgical Treatment Unit for Obesity and Metabolic Diseases” (englobing different medical and surgical specialties such as General Surgery; Internal Medicine; Endocrinology; Anesthesiology; Nutrition; Clinical Psychology; Physical Medicine and Rehabilitation; Gastroenterology; Plastic Surgery). All the participants were informed about the study, and verbal informed consent was obtained.

All the patients involved in the obesity and metabolic diseases program were enrolled in this study. The inclusion criteria for entering the obesity program were the following: age superior to 18 years; BMI ≥ 35Kg/m² with comorbidities or ≥ 40Kg/m² without comorbidities; more than two years of obesity-resistant to conservative medical treatment and educational/behavioral intervention.

Participants completed a Clinical Questionnaire to characterize their pain complaints regarding multiple musculoskeletal locations, characterizing the anatomic location and pain intensity in the last three months.

The population in the study was characterized namely regarding personal feature (gender, age and body mass index) and their pain complaints.

The patients completed a questionnaire referring the numeric rating scale [17] regarding pain in the following seven anatomical regions: hand & wrist, shoulder, lumbar region, hip, knee, ankle and foot. The authors considered the overall score pain to be equivalent to the highest reported pain per region by the patient, experienced in the last three months. The numeric rating scale, ranging from 0 to 10, was used to assess pain intensity in the seven anatomical regions. All the assessments reported were pre-surgery.

Statistical analysis was done using the Statistical Package for Social Sciences (SPSS), version 25.0 (IBM Corp., Armonk, NY). Statistical significance was determined at the level of P < 0.05. In order to assess the normal distribution of the continuous variables, Shapiro-Wilk Test was conducted. Spearman’s correlation coefficient (rho) was used to assess the correlation between the body mass index score and the level of pain per region. Cohen’s standard was used to analyze the correlation coefficients regarding the strength of the relationship [18].

## Results

A total of 466 (342 female (73.4 %)) patients were analyzed. The mean age was 46.28 ± 10.59 years (female: 46.01 ± 10.59; male: 47.02 ± 10.61). The mean participants BMI was 42.77 ± 5.64 kg/m² (females: 42.52 ± 10.51; males: 43.44 ± 5.94), as shown in Table 1.

**Table 1:** Characteristics of participants.

Variable	Group (N = 466)
Female Gender, N (%)	342 (73.4)
Age in years, mean (SD)	46.28 (10.59)
BMI in kg/m², mean (SD)	42.77 (5.64)
Female (SD)	42.52 (10.51)
Male (SD)	43.44 (5.94)

Level of pain by gender

**Figure 1:**
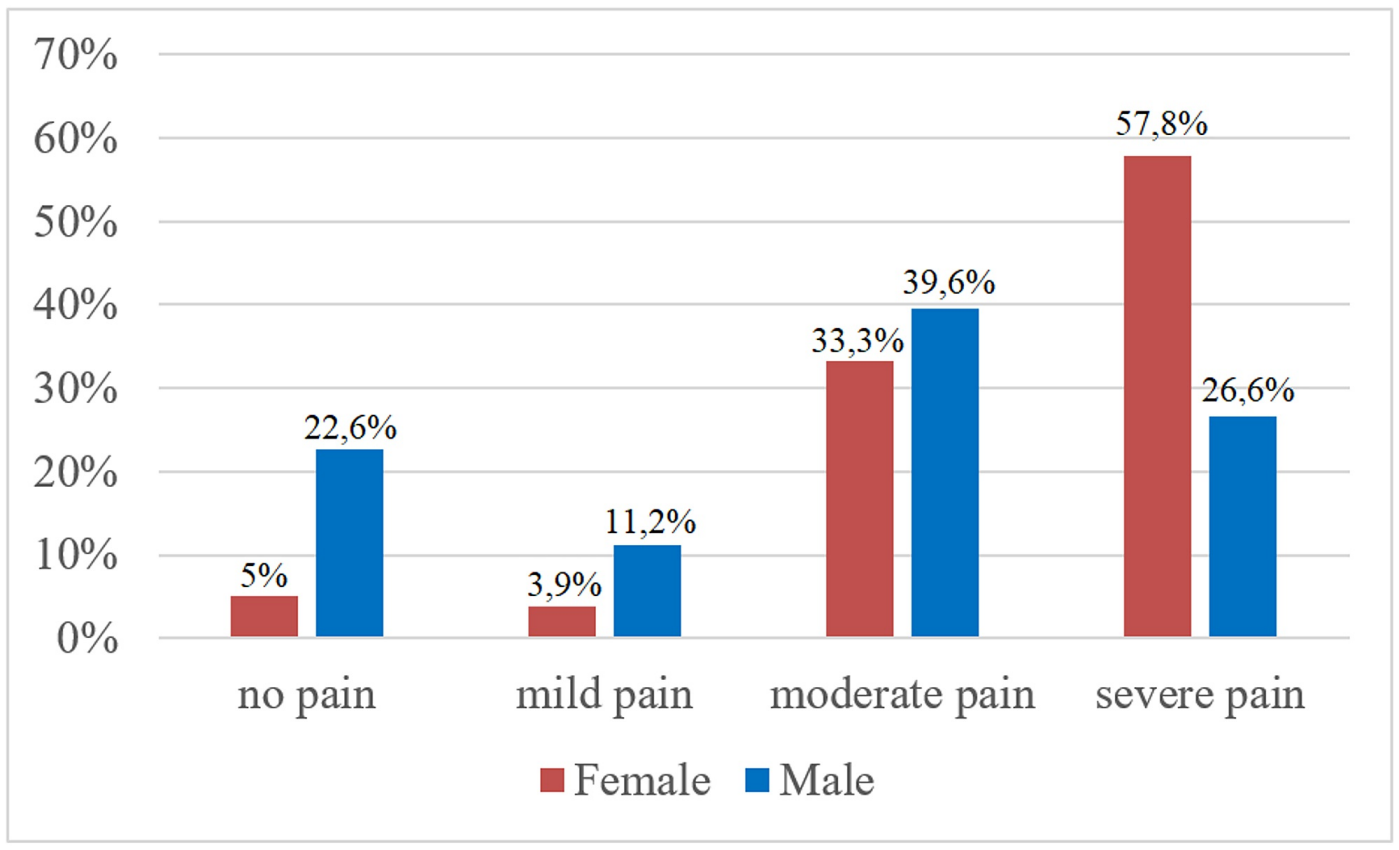
Prevalence and intensity of pain by gender.

The overall prevalence of pain was 90,3%, female 95% vs male 77,4% (P < 0,001). Severe pain was reported by 57,8% of female vs 26,6% of male participants (Figure 1).

Female participants showed a higher mean pain level when compared with male participants (female 6.57 ± 2.38 vs male 4.34 ± 2.89) (P < 0,001).

Prevalence of pain by body region

The prevalence of pain by body region were: hand & wrist 10.5% (N = 49), shoulder 15.7% (N = 73), lumbar region 46.8% (N = 218), hip 29% (N = 135), knee 63.5% (N = 295), ankle 23% (N = 107) and foot 12.4% (N = 58).

Correlation between pain by body region and BMI

BMI showed a moderate positive correlation to the numeric rating scale score in female participants in three body regions: shoulder (rho = 0.440, P = 0.010), knee (rho = 0.410, P = 0.042) and ankle (rho = 0.422, P = 0.024). No statistically significant associations were found in other body regions in both female and male patients, as presented in Table 2.

**Table 2:** Correlation coefficients between pain by body region and BMI.

	Body mass index (BMI)			
	Female		Male	
	Spearman correlation	P	Spearman correlation	P
Hand & wrist	0.016	0.769	0.068	0.455
Shoulder	0.440	0.010	0.005	0.959
Lumbar region	0.004	0.942	0.040	0.660
Hip	0.052	0.336	0.089	0.328
Knee	0.410	0.042	0.013	0.888
Ankle	0.422	0.024	0.057	0.533
Foot	0.023	0.677	0.123	0.174

## Discussion

Results from the current study showed a high MSK pain prevalence among the subjects (90,3%), more in females. An even higher prevalence was found in another study, in which 100% of the obese individuals evaluated during preoperative bariatric surgery reported MSK pain in at least one region of the body [19,20].

Body sites with a higher prevalence of pain were seen especially in areas of increased mechanical load: knee (63.5%), lumbar region (46.8%), hip (29%) and ankle (23%), with a predominance of severe pain. Larsson’s results showed that obese women had more MSK pain in the lower back, knee and foot [21]. A meta-analysis published in 2010 showed that obesity was strongly associated with an increased need for health care, for the treatment of acute and chronic lower back pain [22].

Female participants have reported more pain and a higher prevalence of moderate and intense pain when compared to male participants, as described by other studies reporting that female sex and obesity were factors significantly associated with the persistence or development of MSK pain [23,24], and with higher intensity [25].

Increased BMI ratio was significantly associated with MSK pain among women in shoulder, knee and ankle. It has been demonstrated that overweight is associated with adverse effects on the knees and feet, such as pain, stiffness and functional changes [26,27]. To provide preventive measures and therapeutic interventions for MSK pain, it is crucial to understand the relationship between obesity and pain. The proinflammatory effect of obesity in the pathogenesis of MSK diseases, independent of its biomechanical effect, has also been gaining interest [28]. A systematic review has shown a dose-response relationship between BMI and incident shoulder pain: overweight was associated with an increase of inflammatory factors, which could therefore be related to the development of shoulder disorders [29].

One of the limitations of this study is that the sample selection can contribute to an overestimation of the individuals’ probability of presenting MSK pain, since it is a population with a very high BMI. It is important to acknowledge that severe obesity increases the risk of developing MSK disorders [30].

The study remains ongoing - follow-up after bariatric surgery - to infer whether the expected weight loss is followed by a proportional decrease in pain complaints.

## Conclusions

In conclusion, the authors found a high prevalence of MSK pain and intense pain among the patients, especially in the knee and lumbar regions. Persistent MSK pain is a significant problem in obese patients that can have a negative impact on the quality of life and function. This fact highlights the importance that obese subjects may adopt a preventive healthcare and may have access to an interdisciplinary care, to ensure a more effective intervention and rehabilitation, preventing MSK disorders. More robust studies are needed to detail and further investigate these associations.
